# Digital Dissemination Strategies for Health Promotion Videos in Indigenous Communities in California: Protocol for a Three-Arm Comparative Study

**DOI:** 10.2196/84674

**Published:** 2026-03-17

**Authors:** Lucía Abascal Miguel, Anna E Epperson, Alison B Comfort, Darío León, Mary E Garcia, Alicia R Riley, Nadia Diamond-Smith

**Affiliations:** 1 Institute for Global Health Sciences, University of California, San Francisco, 550 16th St, San Francisco, CA, 94158, United States, 1 (415) 353-7192; 2 Psychological Sciences and Health Sciences Research Institute, University of California, Merced, Merced, CA, United States; 3 Department of Obstetrics, Gynecology and Reproductive Sciences, University of California, San Francisco, San Francisco, CA, United States; 4 Department of Sociology, University of California, Santa Cruz, Santa Cruz, CA, United States; 5 Department of Epidemiology and Biostatistics, University of California, San Francisco, San Francisco, CA, United States

**Keywords:** health communication, vaccine interventions, social media recruitment, Indigenous populations, native American populations

## Abstract

**Background:**

Despite the availability of effective vaccines, flu and COVID-19 uptake remains suboptimal, including among Indigenous communities in California who face unique barriers to accessing public health information. While previous research has evaluated health communication message content and design, fewer studies have systematically compared different dissemination strategies for the same intervention, leaving gaps in understanding optimal approaches for reaching marginalized populations.

**Objective:**

This protocol describes a three-arm dissemination study designed to compare the effectiveness of different strategies for distributing COVID-19 and flu vaccine promotion videos among Indigenous Peoples residing in California, including American Indian and Alaska Natives, and migrant Indigenous Peoples from Latin America.

**Methods:**

Following extensive formative work, including cross-sectional surveys, social network analyses, a discrete choice experiment, and qualitative focus groups conducted with guidance from an Indigenous Community Advisory Board, we developed four 30-second vaccine promotion videos available in English and Spanish, two targeting American Indian and Alaska Native communities and 2 targeting Latin American communities. Videos will be translated to four Indigenous languages (Purépecha, Mam, Zapoteco, and Mixteco). The study compared three dissemination strategies implemented over a two-month period: (1) targeted social media advertisements on Facebook and Instagram, (2) distribution through community-based organizations using their established communication channels, and (3) peer-to-peer sharing through Indigenous community members (“seeds”) recruited from previous research. Primary outcomes focus on dissemination effectiveness, defined by measures of reach (eg, link shares, clicks and video views), engagement (eg, watch time and survey completion), and message impact (eg, trust in the message and vaccination intent), assessed using Bitly link tracking, YouTube analytics, and follow-up surveys. Descriptive and comparative analyses will be conducted to assess differences across dissemination strategies.

**Results:**

The study was funded in January 2023. Intervention development was completed in July 2025. Dissemination activities and data collection across all three study arms were conducted between October 6, 2025, to November 28, 2025, aligned with the COVID-19 and influenza vaccination season in the United States. Data consolidation, cleaning, and analyses began January 2026 and primary results are expected to be submitted for publication in Spring 2026.

**Conclusions:**

This study addresses a critical gap in health communication research by providing a systematic methodology for comparing digital dissemination strategies within Indigenous communities. The combination of community-informed recruitment, multilingual accessibility, and comprehensive digital tracking tools offers a replicable model for evaluating how public health messages spread through different channels, particularly for populations historically excluded from traditional outreach efforts.

## Introduction

Flu and COVID-19 continue to be leading causes of hospitalization and death, particularly for people 65 years of age and older [1]. Despite the availability of vaccines, uptake has stalled in many communities, including in California [2]. As public health communications become increasingly digital, there is a growing need to understand not only what messages are effective, but how those messages are disseminated and received across diverse population.

A growing body of research has examined the design and content of digital health messages, including message framing, visual style, messenger characteristics, and tone, often using social media analytics or survey-based metrics [3-6]. Within this literature, dissemination has increasingly been identified as a critical determinant of communication effectiveness, influencing who is reached, how messages are engaged with, and whether information is trusted and acted upon [7,8]. However, most existing work evaluates dissemination strategies in isolation or confuses other message attributes with delivery strategy, limiting the ability to study their independent contribution to message effectiveness [9,10]. This gap is particularly salient for Indigenous populations, for whom trust, relational networks, and community-based information channels often play a central role in how health information circulates [11,12]. While community-engaged dissemination and peer-led approaches have been highlighted as promising strategies for Indigenous and other marginalized communities, few studies have directly compared multiple digital dissemination strategies using identical content to assess their relative effectiveness [7,13].

This study addresses an important methodological and knowledge gap by comparing three dissemination strategies using identical, community-informed vaccine promotion content across Indigenous populations in California. We use the term Indigenous to refer to American Indian, Alaska Native, Native American, and migrant Indigenous Peoples from Latin America. US census data estimate that approximately 650,000‐760,000 individuals in California identify as American Indian or Alaska Native [14]. Population estimates for Indigenous Peoples from Latin America are limited due to census classification practices; community sources suggest roughly 170,000 may reside in California, though this is likely an underestimate [15].

In this paper, we describe the methods for measuring and testing different dissemination approaches for COVID-19 and flu vaccine promotion videos while holding message content constant. The approaches were selected based on prior dissemination and implementation literature and extensive formative work conducted with Indigenous communities (described below), these are: (1) targeted social media advertisements, (2) through community-based organizations (CBOs), and (3) peer-to-peer sharing through community members (“seeds”).

This dissemination study is the final part of a collaborative 4-year project (2022‐2026) that aims to increase flu and COVID-19 vaccine confidence on Indigenous communities in California, including from Tribal communities with historic lands in the United States and Indigenous migrant communities from Latin America. Three University of California campuses have participated including UC San Francisco, UC Merced and UC Santa Cruz. This research has been guided by an Indigenous Community Advisory Board (ICAB) comprised of Indigenous leaders in their communities and draws from principles of implementation science, human centered design and social network theory. Our formative work included:

1. A cross-sectional survey of health information, vaccine hesitancy, and social media platform use among our target population recruited through social media and our community-based partners completed between 2023 and 2024 [16]
2. Social network analyses using an egocentric network approach to collect data from individuals (ie, *egos*) about the individuals they rely on for health advice and information (ie, *alters*) to identify who within their health network matters in influencing future vaccine intentions (eg, family, friends, healthcare worker), what modes of communication between them are most important (eg, social media, in person) and other network characteristics like trust in these individuals. Our analyses also identified the importance of network members’ experiences with COVID-19, including being supportive of vaccination, encouraging vaccination, having been sick with COVID-19 and having been vaccinated, completed in 2025. (Comfort A, PhD, unpublished data, December 2025)
3. A discrete choice experiment to evaluate preferences for visual and narrative characteristics of posts on vaccines, including other communication attributes (eg, cartoon vs real-life images, tone, messenger) [17]
4. Qualitative focus groups (n=4) with Indigenous adults in California, recruited through our community partners. They were conducted in English, Spanish, and Purépecha, to explore experiences with COVID-19, vaccination, health information access, and social media use, and to contextualize findings from quantitative components, completed between 2024 and 2025. (Riley A, Epperson A, unpublished data, February 2026)

In this study we aim to compare the effectiveness and reach of different digital delivery strategies for disseminating identical, community-informed COVID-19 and influenza vaccine promotion videos among Indigenous populations in California.

## Methods

## Overview

**Figure 1.**
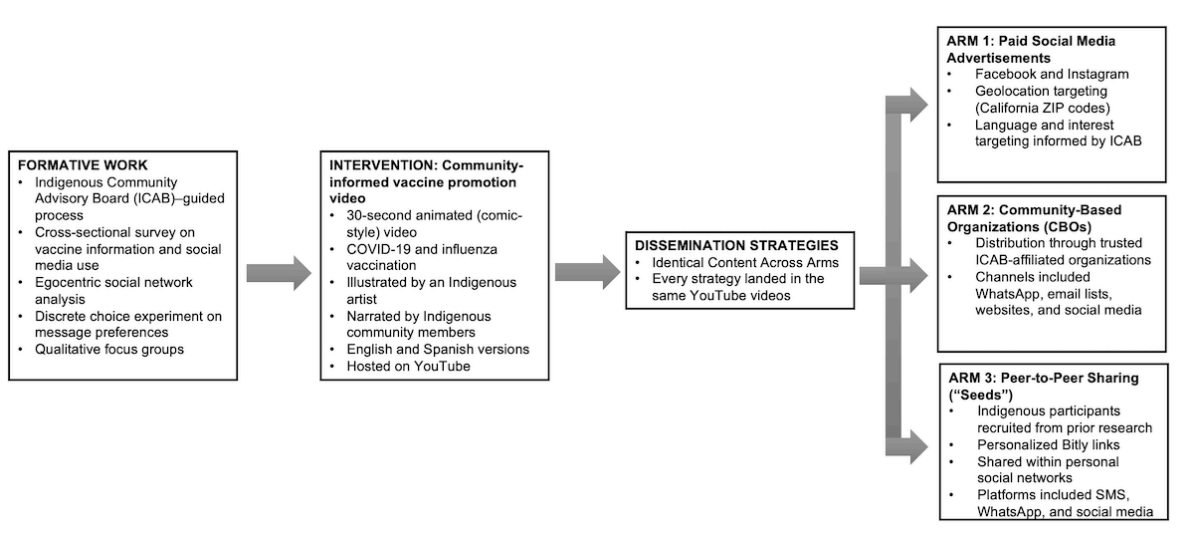
Overview of study design and dissemination strategies.

This study used a three-arm comparative dissemination design to evaluate different strategies for distributing identical COVID-19 and influenza vaccine promotion videos among Indigenous communities in California (Figure 1). The study was conducted during the 2025 COVID-19 and influenza vaccination season in the United States. A table summarizing the study timeline and schedule of dissemination and assessments is provided in Table 1.

**Table 1.** Study timeline and schedule of activities and assessments.

Study phase	Timeframe	Key activities	Data collected
Formative research	2023‐2025	Cross-sectional survey, egocentric social network analysis, discrete choice experiment, qualitative focus groups, ICAB^a^ consultation	Vaccine attitudes, social media use, network characteristics, message preferences
Intervention development	Jan–Jul 2025	Co-creation of vaccine promotion videos with ICAB, illustration and narration by Indigenous artists and community members, translation into Spanish and Indigenous languages	Not applicable
Dissemination period	Oct 6–Nov 28, 2025	Distribution of identical videos through three dissemination arms (paid social media ads, CBO^b^ distribution, peer-to-peer “seeds”)	Bitly link metrics; YouTube reach and engagement metrics
Post-viewing survey	Oct–Nov 2025	Optional survey linked at end of video	Trust in message, vaccination intent, reactions to video, self-reported sharing
Data consolidation and analysis	Jan 2026–present	Cleaning and integration of Bitly, YouTube, and survey data	Primary and secondary outcome measures
Planned dissemination of results	Spring 2026	Manuscript preparation and submission	Not applicable

a ICAB: Indigenous Community Advisory Board.

b CBO: community-based organizations.

### Intervention Development and Design

Guided by our formative findings and in close collaboration with our ICAB, we developed two 30-second vaccine promotion videos. The videos were co-developed through participatory workshops with ICAB members and were produced in English, Spanish, and four Indigenous languages (Purépecha, Mam, Zapoteco, and Mixteco). For this dissemination study, we tested the English and Spanish versions of one video that features family members narrating their decision to receive COVID-19 and influenza vaccines, motivated by prior illness experiences and concerns about protecting their family. The narrative concludes with family members choosing to get vaccinated together.

The videos use a comic-style visual format illustrated by an Indigenous artist and are narrated by Indigenous community members. The two versions are identical in narrative structure, pacing, and messaging, with minor visual adaptations (eg, clothing and color schemes) to reflect either American Indian and Alaska Native or Indigenous Latin American communities, only the English and Spanish versions were used for testing.

The final intervention was implemented between October 6, 2025, to November 28, 2025, timed for the COVID/flu vaccine season in the United States.

### Study Design and Dissemination Arms

In our trial, we will test 3 dissemination strategies for our intervention. The videos will be hosted on our university’s institutional YouTube channel, with separate versions in each focal language. Videos will be distributed through the following three dissemination approaches for one month:

1. Paid social media ads: videos will be shared through a paid ad campaign through Facebook and Instagram. They will be shared as posts with a link to the YouTube video. The audience will be targeted using geolocation through zip codes with high Indigenous populations in California (based on ICAB and census data), language including Spanish, and interests identified with our ICAB.
2. Community based organizations (CBOs): ICAB-affiliated CBOs will share the links to the videos using their usual communication channels used with their members (eg, WhatsApp, social media, email lists, CBO websites).
3. Individual seeds: We will recruit Indigenous-identifying participants from our previous survey (listed above) who consented to future contact. Each individual will receive a link and be asked to share the video with five or more contacts in their network using their preferred method (eg, SMS, WhatsApp, social media).

### Data Collection

#### Outcome Measurement

Outcomes include dissemination performance and message impact across dissemination strategies. The primary outcomes were (1) dissemination reach, measured using platform-level analytics and link-level tracking, and (2) message trust and vaccination intent, assessed through a brief post-viewing survey. Secondary outcomes included engagement metrics (eg, views, watch time, and sharing behaviors) and self-reported changes in views or attitudes toward COVID-19 and influenza vaccination following exposure to the video.

#### Data Collection

Data was collected through three complementary methods to capture both dissemination performance and message impact across strategies:

1. Survey: a clickable Qualtrics survey link was shared at the end of the video to collect post-intervention data. The brief survey assessed likelihood of flu and COVID-19 vaccination, reactions to the video, how the video was shared, and trust and engagement with the video. Participants who completed the survey were entered into a gift card raffle.
2. Link dissemination tracking: Bitly was used to create individual and personalized links for each dissemination strategy (eg bit.ly/socialmedia). We made a single link for all social media ads, one link per organization for the participating CBOs; and one link per participant for the seeds. We used Bitly analytics to track link engagement, clicks, locations, referrals and type of devices used. All links will corresponded to the same YouTube videos.
3. YouTube analytics: we used YouTube metrics to track platform-level spread and engagement including video reach metrics (impressions, click through rate, views, etc), engagement metrics (watch time, retention, likes, comments, shares), audience insights (age and gender, viewers) and follow-ups (end screen and cards).

### Analysis

We will conduct both descriptive and comparative analyses to evaluate the effectiveness of each dissemination strategy. Dissemination patterns within each arm will be summarized, including views, link interactions, and survey responses. Where possible, metrics will be standardized (eg, engagement per view or per dissemination unit) to compare across strategies that could have different scales of deliveries.

Differences between arms will be assessed using aggregated platform (YouTube)- and link-level (Bitly) metrics, as well as survey-based outcomes. For survey measures of trust in the video and vaccination intent, distributions will be compared across dissemination strategies using logistical regressions controlled for sociodemographic characteristics (age, gender, and race/ethnicity). Subgroup analyses (eg, by age group, including adults aged ≥65 y) will be conducted where feasible and will depend on the overall sample size and the distribution of participants within each subgroup.

We will examine indicators of dissemination efficiency, such as reach per dissemination unit (eg, per organization, per seed, or per advertising campaign) and engagement. These comparisons are intended to identify dissemination approaches with greater potential for scalability and sustainability in Indigenous communities. The study is not powered to detect predefined effect sizes. Instead, results will provide empirical benchmarks to guide dissemination strategy selection in larger implementation efforts.

### Ethical Considerations

This trial was registered at ClinicalTrials.gov (NCT07096245) and approved by the University of California, San Francisco Institutional Review Board (IRB #23‐38709). No personal or identifiable data were collected through YouTube, Bitly, Facebook, or Instagram analytics; these platforms were used solely to obtain aggregate dissemination and engagement metrics. All survey data were collected through a secure UCSF (University of California, San Francisco) Qualtrics account. Participants who completed the post-viewing survey provided electronic informed consent prior to participation and, if interested, were entered into a raffle for one of forty US $100 gift cards. Individuals recruited as peer-to-peer “seeds” were consented in advance and received $30 in compensation for distributing the intervention content within their networks.

## Results

The study was funded in January 2023. Intervention development, including formative work and the co-creation and translation of vaccine promotion videos, was completed in July 2025. Dissemination activities and participant-facing data collection across all three study arms were conducted between October 6 and November 28, 2025, aligned with the COVID-19 and influenza vaccination season in the United States (Table 1).

Three dissemination strategies were implemented during this period. For the community-based organization arm, five community-based organizations participated in distributing the video links to their members using established communication channels. For the peer-to-peer dissemination arm, 68 Indigenous-identifying participants from prior research were recruited as individual “seeds” and provided with personalized links to share the videos within their social networks. In parallel, targeted paid social media campaigns were conducted on Facebook and Instagram using geolocation, language, and interest-based targeting strategies informed by formative research and guidance from the ICAB.

All dissemination activities concluded on November 28, 2025. Data from Bitly link tracking, YouTube analytics, and follow-up surveys were consolidated and prepared for analysis beginning in January 2026. Outcome analyses are underway, and primary results are expected to be submitted for publication in Spring 2026.

## Discussion

This study was designed to compare how identical, community-informed vaccine promotion content performs when disseminated through different digital channels among Indigenous communities in California. We hypothesize that dissemination strategies that leverage trusted relationships, such as community-based organizations and peer-to-peer sharing, will demonstrate higher levels of trust and message impact compared to standard social media advertising, while paid ads may achieve greater overall reach.

As public health communication continues to shift into digital spaces, and as people rely on a growing number of virtual platforms to connect, it becomes more important to understand how health messages are shared and how the messenger impacts how people trust those messages. This project responds to a gap in health research by testing three different strategies to distribute the same vaccine information video to Indigenous communities in California, communities that have often been left out of traditional outreach. We compare broad social media ads, which are commonly used today, to more targeted approaches through trusted community organizations and through individual participants sharing within their own networks.

One of the strengths of this study is the combination of digital tracking tools, like Bitly and YouTube, with a post-intervention survey. This allows us to see how messages move across systems and gives us a way to compare which strategy leads to more views, engagement, and trust. These insights can help shape future campaigns by showing how public health information can be distributed in ways that are more meaningful and relevant for the people they aim to reach. In addition, we ensure accessibility of the message by making it available in four Indigenous languages spoken in California.

We do expect some limitations. For example, the success of the seed-sharing strategy depends on whether participants decide to share the video, and whether the people they share it with choose to watch it and respond to the survey. These steps are voluntary, and it is anticipated that there may be some participant drop-off.

This study will provide a practical and grounded model for testing how digital public health communication interventions are shared, especially when the goal is to reach communities who are not usually centered in public health campaigns, who may have less trust in government resources due to historical experiences, and who may be harder to reach or have other barriers (language, etc).

## Abbreviations

CBO: Community-based organization
ICAB: Indigenous Community Advisory Board
IM: Indigenous migrant
IRB: Institutional Review Board
UCSF: University of California, San Francisco
